# Supplementary material for: Predicting chromatin conformation contact maps
Source: bioRxiv. 2024 Apr 14:2024.04.12.589240. Preprint. [Version 1] doi: 10.1101/2024.04.12.589240 (PMC11030330; doi:10.1101/2024.04.12.589240)
Supplement: Supplement 1 [file media-1.pdf]

Rank	Loss	Assay	Cell type	Position	Layers	Nodes	Dropout	Learning Rate
1	0.0523	128	16	128	4	256	0.4	0.0005
2	0.0524	64	64	512	6	1024	0.2	0.0050
3	0.0527	64	128	128	4	1024	0.2	0.0050
4	0.0529	128	16	1024	4	256	0.2	0.0005
5	0.0531	256	256	128	6	1024	0.4	0.0005
6	0.0534	16	256	1024	2	1024	0.2	0.0005
7	0.0538	128	16	128	8	1024	0.4	0.0005
8	0.0539	256	128	128	4	256	0.2	0.0050
9	0.0540	128	64	256	4	256	0.6	0.0005

Table S1: **Results of the hyperparameter search.** The table lists the best-performing nine hyperparameter settings, with the corresponding loss values.

Biosource	Assay Type	Accession	Generating Lab
192627	Dilution Hi-C	4DNESYPKLMAM	Erez Lieberman Aiden
192627	in situ Hi-C	4DNESECNR4O8	Erez Lieberman Aiden
CC-2551	Dilution Hi-C	4DNESUB35TII	Erez Lieberman Aiden
CC-2551	in situ Hi-C	4DNESIE5R9HS	Erez Lieberman Aiden
GM12878	Dilution Hi-C	4DNESLLTENG9	Bing Ren
GM12878	DNA SPRITE	4DNESI1U7ZW9	Mitchell Guttman
GM12878	in situ ChIA-PET CTCF protein	4DNES7IB5LY9	Yijun Ruan
GM12878	in situ ChIA-PET RNA Pol II	4DNESZ25MOZV	Yijun Ruan
GM12878	in situ Hi-C	4DNESPXW8XHY	Erez Lieberman Aiden
GM12878	PLAC-seq H3K4me3	4DNESL3LFLGI	Bing Ren
H1-hESC	in situ ChIA-PET CTCF protein	4DNESR9S8R38	Yijun Ruan
H1-hESC	in situ ChIA-PET RNA Pol II	4DNESNYUGLUN	Yijun Ruan
H1-hESC	in situ Hi-C	4DNES2M5JIGV	Job Dekker
H1-hESC	Micro-C	4DNES21D8SP8	Job Dekker
H1-hESC	PLAC-seq H3K4me3	4DNESQMO66LZ	Bing Ren
HeLa cell line	Dilution Hi-C	4DNESWMJBQMR	Jan-Michael Peters
HeLa cell line	DNase Hi-C	4DNESGEEV6TJ	Todd Waldman
HeLa cell line	in situ Hi-C	4DNESEDV9YMX	Job Dekker
HeLa cell line	Micro-C	4DNESA5PN8AB	Job Dekker
HFF-hTERT	Dilution Hi-C	4DNES9L4AK6Q	Job Dekker
HFF-hTERT	in situ Hi-C	4DNESB6MNCFE	Job Dekker
HFF-hTERT	Micro-C	4DNESEGKQY7I	Job Dekker
HFFc6 (Tier 1)	DNA SPRITE	4DNESJYGTI8S	Mitchell Guttman
HFFc6 (Tier 1)	in situ ChIA-PET CTCF protein	4DNESCQ7ZD21	Yijun Ruan
HFFc6 (Tier 1)	in situ ChIA-PET RNA Pol II	4DNESI1WZ5HT	Yijun Ruan
HFFc6 (Tier 1)	in situ Hi-C	4DNES2R6PUEK	Job Dekker
HFFc6 (Tier 1)	Micro-C	4DNESWST3UBH	Job Dekker
HFFc6 (Tier 1)	PLAC-seq H3K4me3	4DNESIF5UIQE	Bing Ren
HUVEC cell	Dilution Hi-C	4DNESOSE2FYZ	Erez Lieberman Aiden
HUVEC cell	in situ Hi-C	4DNESEW5JLUC	Erez Lieberman Aiden
IMR-90	Dilution Hi-C	4DNESSM1H92K	Erez Lieberman Aiden
IMR-90	in situ Hi-C	4DNES1ZEJNRU	Erez Lieberman Aiden
WTC-11	in situ ChIA-PET CTCF protein	4DNES8MZ76GP	Yijun Ruan
WTC-11	in situ ChIA-PET RNA Pol II	4DNESRRTL4BU	Yijun Ruan
WTC-11	in situ Hi-C	4DNESPDEZNWX	Job Dekker
WTC-11	Micro-C	4DNESODGV2V2	Job Dekker
WTC-11	PLAC-seq H3K4me3	4DNESDRL4ZKM	Bing Ren
WTC-11 AAVS1-GFP C28	DNase Hi-C	4DNES8BLXVP5	Chuck Murry
WTC-11 AAVS1-GFP C28	in situ Hi-C	4DNESJ7S5NDJ	Job Dekker
WTC-11 AAVS1-GFP C28	Micro-C	4DNESAGG7EUC	Job Dekker
WTC-11 AAVS1-GFP C28	PLAC-seq H3K4me3	4DNESIZ5TTHO	Bing Ren

Table S2: **4D Nucleome data sets used in this study.**

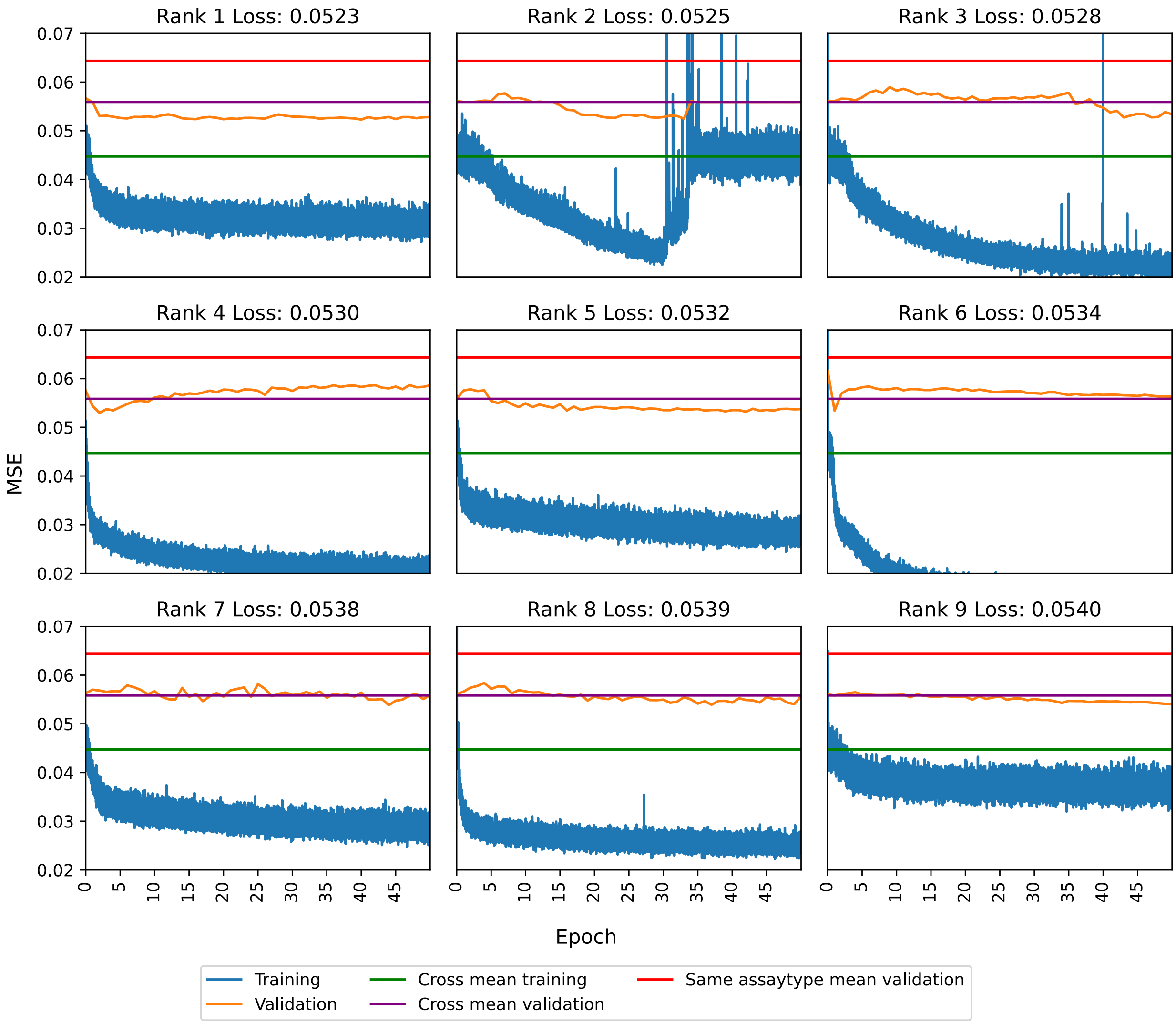

Figure S1: **Loss curves demonstrate sufficient training epochs.** The loss curves for the 9 lowest hyperparameter combinations are shown. The associated hyperparameter combinations are shown in Table S1. The Sphinx training loss (blue), Sphinx validation loss (orange), cross-mean baseline training loss (green), cross-mean validation loss (purple), and same-assay validation loss (red) are shown.
